# Focus on self-healing materials: recent challenges and innovations

**DOI:** 10.1080/14686996.2021.1888528

**Published:** 2021-03-15

**Authors:** Wataru Nakao, Toshio Osada, Tomoya Nishiwaki, Hideyuki Otsuka

**Affiliations:** Faculty of Engineering, Yokohama National University, Yokohama, Japan; Research Center for Structural Materials, National Institute for Materials Science, Tsukuba, Japan, OSADA.Toshio@nims.go.jp; Department of Architecture and Building Science, Tohoku University, Sendai, Japan; Department of Chemical Science and Engineering, Tokyo Institute of Technology, Tokyo, Japan

One of the material researchers’ dreams is to create artificial materials that have the same functions as natural materials, such as living things. Self-healing material is a material that has a function found in organisms, and naturally exhibits extremely high robust performance. Owing to such a dream function, self-healing materials have been independently researched and developed for each particular material system, such as metal, ceramics, concrete, polymer, and composite material. There is a long history of the research on self-healing materials. For example, self-healing ceramics were first reported in the 1960s. Tracing back with the development of stainless steel to 1904, we would notice that stainless steel could also be considered as one of the self-healing materials, as its oxidation protection layer is spontaneously repaired. Going even further back in history, the first report of self-healing concrete by the precipitation of calcium carbonate can be found as early as 1836.

The year 2007 marked a major turning point for the research of self-healing materials. In that year, the first international conference on self-healing materials was held, and it was led by Professor van der Zwaag (Delft University of Technology, The Netherlands) and Professor White (University of Illinois, USA). The most important topic of the conference was to create a concept for common self-healing materials that can be applied in all materials. This discussion has greatly facilitated the exchange of knowledge and accelerated the research on self-healing materials. The 7th International Conference on Self-healing Materials was held in 2019 (Yokohama, Japan), which triggered the creation of this focus issue.

This focus issue covers various types of self-healing materials, with special focus on latest research results of self-healing materials that were developed after the turning point. Research papers introduce the diverse and recent research topics regarding novel mechanisms, design, modelling, computational simulations, and materials standardization for applications. In addition, the review paper that introduces recent progress in self-healable ion gels will be useful for understanding the background and prospects of self-healing materials. This special issue of *Science and Technology of Advanced Materials* will be helpful for scientists who want to gain the basic understandings of self-healing materials and to start their own original research on self-healing materials. Finally, we sincerely hope that this focus issue contributes to accelerating research, development, and future applications of advanced self-healing materials.

